# Suicidal behavior in problematic substance uses in South Gondar zone, Northwest Ethiopia: a cross-sectional survey

**DOI:** 10.1186/s13011-020-00303-4

**Published:** 2020-08-18

**Authors:** Getasew Legas, Habte Belete, Sintayehu Asnakew, Amsalu Belete, Shegaye Shumet

**Affiliations:** 1 Department of psychiatry, College of Medicine and Health Sciences, Debre Tabor University, Debre Tabor, Ethiopia; 2 Department of psychiatry, College of Medicine and Health Sciences, Bahir Dar University, Bahir Dar, Ethiopia; 3 Department of psychiatry, College of Medicine and Health Sciences, Gondar University, Gondar, Ethiopia

**Keywords:** Suicidal behavior, Risk factors, Problematic substance use, Low-income country

## Abstract

**Background:**

Suicidal behavior has a significant contribution to the global burden of disease that affects individuals, families and communities at different age groups. Sadly, up to 75% of suicides in the world occur in low-and- middle income countries which have no adequate resource to prevent it. The aim of this study was to assess suicidal behavior and associated factors among community residents with problematic substance use in South Gondar zone, northwest Ethiopia.

**Methods:**

Community based cross-sectional survey was conducted by using a suicidal behavior revised questionnaire from January 15 to March 15, 2019. A total of 4035 participants were screened for problematic substance use by using multi stage cluster sampling and 846 participants were positive for problematic substance use then asked for suicidal behavior. Multiple logistic regression analyses used to see adjusted odd rations (AOR). Multilevel binary logistic regression was used to account for the hierarchical structure of the two-level data within individual and districts level.

**Results:**

The prevalence of suicidal behavior over the last 12 months in problematic substance uses was found to be 41.4% with 95% of confidence interval (CI) (38.2–44.9). Perceived stigma, [AOR = 1.605, 95% CI (1.16–2.23)], family history of suicide [AOR = 3.22, 95% CI (1.46–7.10)], physical illness [AOR = 2.45 95% CI (1.157–3.84)], rural resident [AOR = 1.74, 95% CI (1.16–2.62)], depression [AOR = 4.44, 95% CI (3.15–6.27)] and living alone (AOR = 1.61, 95% CI (1.16–2.24) were risks factors for suicidal behavior.

**Conclusion:**

Suicidal behavior in problematic substance uses found to be high. Health workers should pay attention to decrease suicidal behavior and to control amendable factors.

## Background

Current global burden of disease reports that mental, neurological and substance use disorders were accounted for 258 million disability-adjusted life years (DALYs) and of this, 14% of DALYs were accounted by only substance use disorders [1]. Approximately 1.53 million people will die from suicide yearly and 10–20 times more people attempt suicide globally. On average, in every 20 s one death and in every 1–2 s, one attempt can occur [2] that linked to substance use disorders which responsible for 22.5 million DALYs and contribute to suicide [3].

The 2016 global mental health reports 75% of suicides in the world occur in low- and- middle income countries [4]. From reviews and meta-analyses that published between January 1, 1998 and February 19, 2014 across different countries, neuropsychiatric disorders had the highest risks of mortality than general population in which substance use disorder has found the highest risk of suicide [5]. Other review and controlled psychological autopsy studies in Germany reported that suicide cane occurs 19% up to 63% from substance use disorders and commonly occurs from alcohol use disorders. Suicide is very common in alcohol use disorder and comorbid major depressive disorder [6]. Australian study reports high risk of suicide in heroin users than non-users across different countries [7]. According to World Mental Health Survey reports; female sex, younger age, lower education and income, rural area, unmarried status, unemployment, parent psychopathology and mental disorders were the possible risk factors of suicidal behavior both in developed and developing countries [8].

Magnitude of suicidal behavior is different around the world. From participants with problematic substance use in USA, 34.1% of them attempt suicide at least once whereas comorbid depression was increased the odds of suicidal attempt [9]. Among people with drug addiction in Brazil, 43.9% of drug users had history of suicidal behavior and depressive symptoms, family history of suicide, maternal psychiatric history and smoking were associated with suicidal ideation and attempt [10, 11]. Suicidal behavior reported in 37.6% of participants who had alcohol dependence and in 29.5% of participants who had other drug dependence in USA [12]. In South Africa, 23% of suicide reports due to problematic alcohol use and residents from rural areas were more committed suicide than residents from urban areas whereas mental disorder predicts the onset of suicidal ideation [13, 14]. In Ethiopia, a number of community-based research conducted in the general population and prevalence of alcohol use disorder ranges 12.4 to 21% [15–17].

Even though problematic substance use has been reported 14.1% among students from poly technique college [18]; however, there is no evidence on prevalence of suicidal behavior among individuals with problematic substance uses’ and has a limited published literature on this topic in Ethiopia. Therefore, this study will add a body of knowledge about prevalence and factors associated with suicidal behavior in the community participants with problematic substance use. Assessing and showing the significance of suicidal behavior in substance abuse at the community level is important to enforce policy-makers and different stakeholders to integrate mental health service with primary health care system to manage suicidal behavior of people with problematic substance use at the community level.

## Methods

### Study setting

This study was conducted in South Gondar zone, Amhara region that located in the northwest part of Ethiopia. Debre Tabor town is the capital city of south Gondar zone which is 666 km far from Addis Ababa and 99 km far from Bahir Dar (the capital city of Amhara region). According to the 2007 population census report; the total population size of South Gondar is estimated 2,051,738 and had seven primary and one referral hospitals. Even though alcohol, khat, nicotine (smoking) and cannabis are the most common available substance in South Gondar zone, only three hospitals deliver mental health service for the community.

### Participants, sampling and data collection

All permanent (residing at least 6 months) adult residents of south Gondar zone were the source population whereas those adults whose age 18 years and above were the study population. Participants who were clinically diagnosis for known sever mental illness (who received a clinical diagnosis from mental health professionals or psychiatrists) who cannot give appropriate information due to their psychosis, low energy, motivation/hyperactivity and also difficult to give interview due to complaining of any discomfort or pain, instability were excluded from the study.

The sample size was calculated by using single population proportion by considering 50% proportion of suicidal behavior among people with problematic substance uses (for unknown prevalence) with 5% margin of error, and 95% confidence interval. We added 10% non-response rate and then multiplied by 2 as we used the design effect of 2. The final calculated sample size was 846, but only 804 participants were complete the interview. From 42 non-responded participants, 15 denied to participate; 16 failed to complete the interview and 11 were excluded due to the exclusion criteria (because of unable to communicate due to complaining of discomfort or pain or instability due to their psychopathology).

**Fig. 1:**
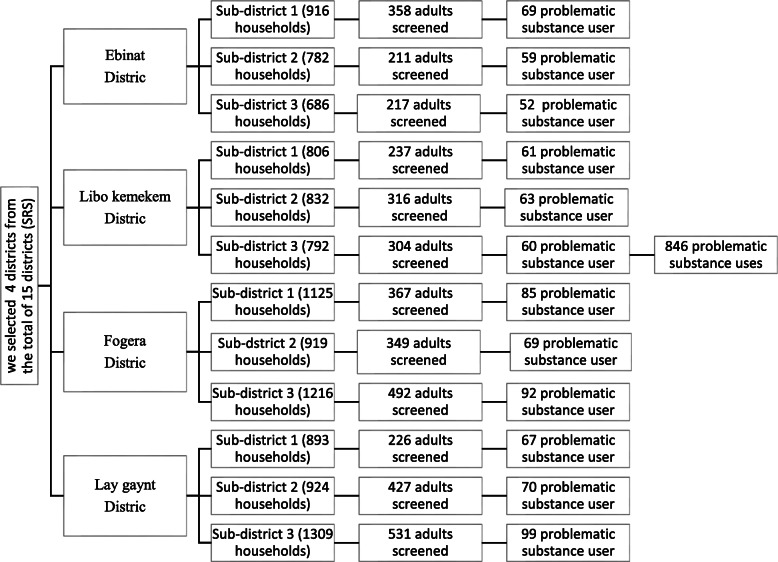
Shows the sampling process and how participants were recruited in the community at different districts. It presents the sampling process from the district to the household level. Four districts out of the total 15 districts are presented in the left side of the graph. Three sub-districts from each of the four districts are presented in the graph that makes total twelve sub-districts. Individual participants were interviewed at the household level in each sub-district and presented in the right side of the graph. The sampling process is described in greater detail in the main text

Multi stage cluster sampling technique was used to select study participants. First, we were select four districts by lottery method from the total of 15 districts and select three sub-districts in each of selected districts. There was a total of an average 11, 200 households in the selected sub-districts and from this, 4035 participants/households (since we included more than one household member if they meet the inclusion criteria in one household) were screened door to door knocking for problematic substance misuses by CAGE AID questionnaire (Cut down, Annoyed, Guilty, and Eye-opener) until we get the determined sample size, 846 (since we had limited on this sample size, we did not include all identified households in the sample, and there are far fewer people screened compared to the number of households identified). Then, we assessed suicidal behavior among 846 participants who were positive for problematic substance use. Figure 1 presents the sampling process, the number of districts included and participants screened in the community. In this figure four districts, 12 sub-districts and 846 household/participants were included the sampling process in South Gondar Zone. We had screened all adults whose age was 18 years and above in each household for problematic substance use until we get the calculated sample size, 846 from 15 January to March 15, 2019. The data was collected by face to face interview. We recruited psychiatry nurses for data collection and the interview was delivered by using a semi-structured questionnaire which was translated into Amharic version (local working language). Participants were recruited by visiting to their home, and that three visits were made if participants were unavailable. The data collectors were visit their home (or next appointment was made) based on the convenient time for the availability of the participant at their home.

### Measurements

Problematic substance use was screened by CAGE AID questionnaire which has 4 questions. If a participant score two or more positive answers from the total of four questions was considered positive for problematic substance use. The CAGE AID has been used in previous Ethiopian studies [18, 19]. A 12 months suicidal behavior was assessed by using suicidal behavior questionnaire revised (SBQ-R). The instrument was adopted for assessing suicidal behavior (ideation, plan and attempt). SBQ-R is a shortened scale that contains four items on suicidal behaviors and ideation over the past 12 months, suicide-related communication, and self-reported likelihood of any future suicidal behavior. The total score ranges from 3 to 18 and we used a scores ≥7 for considering having suicidal behavior for our study that found with a sensitivity of 0.93 and specificity of 0.95 that indicates the risk of suicide in the general adult population [20]. SBQ-R has been also approved as a useful scale for suicide risk assessment in clinical and non-clinical samples [21].

Depression was assessed by using patient health questionnaire (PHQ-9) to classify whether depression is present or not. PHQ-9 has nine items and each item was rated on a four-point scale, 0 (not at all), 1 (several days), 2 (more than half the days) and 3 (nearly every day) with the total score ranging from 0 to 27. To include mild symptoms of depression, we use a score of five or more on PHQ-9 questionnaire to indicate the presence of depressive episode for the last 2 weeks [22]. Perceived Stigma of Substance Abuse Scale was used to assess the individual’s perception about their substance abusive behavior in the community. It has 8 items with four Likert scale from “strongly disagree” to “strongly agree” and the total score ranges from 8 to 32 with higher scores from the mean indicating greater perceived stigma [23]. Social support was assessed by using three items of Oslo social support scale which ranges from three to 14 and scoring of 12–14 “strong support”, score of 9–11 “moderate social support” and score of 3–8 “poor social support” in Oslo-3 social support scale [24].

### Analysis

The data were entered to Epi-data 3.1 and then exported to SPSS version 20 for analysis. The result was present by frequencies/percentages, and odds ratios and adjusted odds ratios had been calculated by using logistic regression. A multilevel binary logistic regression model was applied to represent the odds that a given participant living in a given district would report having suicidal behavior. We used multilevel binary logistic regression to account for the hierarchical structure of the two-level data with individuals (level 1) nested within districts (level 2).

### Ethical clearance

Ethical clearance was obtained from Ethical Review Committee of Debre Tabor University and permission letter was taken from Zone administration. Name and address of the participants was not taken, and participants was informed about the aim, and advantages of the study. Written consent was taken before data collection, and confidentiality and their rights even to stop in the middle of the interview was kept. Referral to psychiatric clinic was given for participants who were screened positive for problematic substance use, suicidal behavior and depression.

## Result

A total of 804 respondents were participated in this study with the response rate of 95%. Majority of the respondents were male 615 (76.5%). Most of the respondents were single 420 (52.2%), Orthodox Christian by religion 720 (89.6%) and Amhara by ethnicity 759 (94.4%) (Table 1). Regarding to their job and residency most participants were jobless and urban residents.

**Table 1:** Socio-demographic characteristics of the participants with problematic substance use (*n* = 804)

Variable	Category	Frequency	Percentage
Age	18–30	342	42.5%
	31–45	282	35.1%
	> 45	180	22.4%
Sex	Male	615	76.5%
	Female	189	23.5%
Ethnicity	Amhara	759	94.4%
	other	45	5.6%
Educational status	Unable to read and write	153	19.1%
	1–8 grade	132	16.4%
	9–12 grade	222	27.6%
	Diploma & above	297	36.7%
Religion	Orthodox Christian	720	89.6%
	Muslim	84	10.4%
Marital status	Single	420	52.2%
	Married &living together	222	27.6%
	Separated	63	7.8%
	Divorced	72	9.0%
	Widowed	27	3.4%
Living circumstance	With family	480	59.7%
	Alone	324	40.3%
Residence	Rural	153	19.0%
	Urban	651	81.0%%
Job status	Jobless	462	57.5%
	Has job	342	42.5%

### Prevalence of suicidal behavior

From the total of 804 problematic substance users 333, 41.4% (95% CI: 38.2, 44.9) of problematic substance users had suicidal behavior over the past 12 months.

### Factors associated with suicidal behavior

To determine the association of independent variables with suicidal behavior, bivariate analysis and multivariable binary logistic regression analysis were carried out. Poor social support, perceived stigma, having comorbid physical illness, living circumstance (living alone), family history of suicide, rural residency and comorbid depression were found associated with suicidal behavior in bivariate analysis. Perceived stigma, having comorbid physical illness, living circumstance (living alone), family history of suicide, rural residency and co morbid depression were associated with a 12 months suicidal behavior in multivariable analysis (Table 2).

**Table 2:** Bivariate and multivariate analysis of 12 months suicidal behavior among problematic substance use respondents in south Gondar zone, northwest Ethiopia 2019

Variables	Category	Suicidal behavior		COR 95% CI	AOR 95% CI	*P*-value
		No	Yes			
Perceived stigma	Yes	239	138	1.456 (1.097–1.932)	1.521 (1.105–2.09) *	**0.010**
	No	232	195	1	1	
Living circumstance	With family	314	166	1	1	
	Alone	157	167	2.012 (1.509–2.683)	1.543 (1.116–2.13) *	**0.008**
Comorbid physical illness	No	422	253	1	1	
	Yes	49	80	2.713 (1.847–4.015)	2.492 (1.604–3.87) *	**0.0001**
Depression	No	368	145	1	1	
	Yes	103	188	4.632 (3.406–6.301)	4.512 (3.226–6.31) *	**0.0001**
Family history of suicide	No	459	303	1	1	
	Yes	12	30	3.787 (1.909–7.513)	2.880 (1.315–6.31) *	**0.008**
Residence	Urban	398	253	1	1	
	Rural	73	80	1.724 (1.210–2.457)	1.811 (1.213–2.71) *	**0.004**

Note: 1 (reference group), * (*p* < 0.05), COR (crude odds ratio), AOR (adjusted odds ratio)

### Multilevel analysis

The results of the multilevel binary logistic regression analyses are reported in Table 3. This examination showed that suicidal behavior was not varied significantly across districts (β = .168, *p* = .279). Model 1 presents the effects of individual-level variables. For the individual-level variables; perceived stigma, comorbid physical illness, living alone, family history of suicide, rural residence and co morbid depression were associated with a 12 months suicidal behavior.

**Table 3:** Multilevel logistic regression analyses of contributing factors to suicidal behavior among participants

Model 1 Fixed effects β				
Variables	Β	P	OR	95% CI
Intercepts	.320	.504	.726	.284–1.859
Stigma	.473	.005	1.605	1.155–2.230
Depression	1.491	<.001	4.442	3.146–6.273
Living alone	.475	.005	1.608	1.156–2.236
Rural residency	.555	.008	1.741	1.158–2.618
Family history of suicide	1.132	.005	3.22	1.46–7.100
Comorbid medical illness	.896	<.001	2.451	1.1566–3.835
Model χ2 =	16.411	*P* < .001		

## Discussion

Suicide prevention becomes an emerging priority for health care across the globe. a number of suicide deaths are reported recently from the community; however, suicide prevention has not given attention as a core priority in the health care system [25]. In this study, the 12 months prevalence of suicidal behavior was found to be 41.4% (95% CI: 38.2, 44.9) among residents with problematic substance use in South Gondar zone, northwest Ethiopia.

This finding was relatively consistent with a study done among people with drug addiction problems in Brazil, 43.9% and a study done in Frankfurt, Germany, 41% [10, 26]. However, the current finding was lower than among those studies conducted in Saudi Arabia and US, where suicidal behavior was reported by 50.7 and 50.1% of people with substance misuse respectively [15, 17]. The current finding also higher when compare with other studies such as in Sweden 33.3% [27], in New Zealand 16.2% [28], In Barcelona Spain 30.8% [29], in Norway 16% [30] South Africa 23% [13], Tanzania 4.6% [31] and a study done in Sub- Saharan Africa (Zambia 31.9%, Kenya 27.9%, Botswana 23.1%, Uganda 19.6%, Tanzania 11.2% [32]. The possible reasons for this prevalence discrepancy might be due to differences in the study population, sample size, utilized instrumental, socioeconomic and cultural differences also take the role.

Among associated factors of suicidal behavior, living alone had 1.6 times risk for suicidal behavior which is supported by a study conducted in young French adults [33] and from systematic review study [34]. This is probably explained in terms of poor social interaction and lack of psychological support can lead to suicidal behavior. People with problematic substance use may face different social limitations to build a positive relationship with the community that lead them to live alone and then may resulted suicidal behavior.

It had found that an association between comorbid physical illness and suicidal behavior among participants with problematic substance use. Suicidal behavior was 2.5 times risk in participants with medical comorbidity than their counterparts. Those who are living with chronic physical illness increases the likelihood of behavioral disorders and may think of ending once life. The dual effect of their behavioral problems and burden of medical illness has become worsen and may push them to have poor quality of life like limited daily activity, experience dissatisfaction in life which may expose them for depression and finally suicidality compared with their healthy counterpart. This finding agrees with studies done in lower income countries [35] and France [33].

Suicidal behavior was 1.7 times more common among participants who live in rural area compared with individual who live in urban area. The result is similar with World Mental Health Survey report and a research done in South Africa [8, 13, 14]. This may be due to rural areas were significantly less likely than those from urban areas to seek professional help for a mental health disorder or perceived need for treatment [36].

The prevalence of suicidal behavior was higher among those who reported having family history of suicide. Individuals with family history of suicide were about three times more likely to have suicidal behavior when compared to individuals who did not reported. This result is in line with a study done in South Africa [37], Brazil [10] and from other systematic review study [38]. The possible reasons may be due to parental psychopathology can increase the risks of suicidal behavior in the offspring through different reasons like transmission of genetic factors associated with increased levels of distress and early environmental stressors due to parental psychopathology [39–42].

Suicidal behavior was more than four times more common among those participants who reported comorbid depression than their counterparts. This finding was supported with studies in USA [43], Brazil [10, 11], France [33] and with other studies [9, 34, 44]. Suicidal behavior is highly linked with depression and possible reason might be comorbid depression might have low level serotonin. The effect of low level of serotonin might have a contribution to feeling guilt, excessive worthlessness and hopelessness that can be resulted suicidal behavior [45].

Perceived stigma due to problematic substance use was 1.6 times more common among those participants with suicidal behavior. Off course, stigma found a risk factor of suicidal behavior which is supported by previous studies in Ethiopia [46, 47] that perceived substance or medical related stigma has a risk for suicidal behavior. Feeling sense of stigmatized by the public may get one’s life wired and boring that results suicidal behavior. However, this result was inconsistent with study in southern Germany [48]. The possible reason might be a result of psychological distress and failure of being accepted by mainstream society [49].

Our findings targeting on the association between problematic substance use disorder, perceived stigma and related suicidal behavior is its strength which is rare in previous studies in Ethiopia.

We have a number of limitations, and low sample size at zonal level may affect the generalizability of the finding and low proportion of female participants may affect the representativeness of the results.

## Conclusion

The 12 months prevalence of suicidal behavior among problematic substance uses was high which needs public health attention and emphasis. Biopsychosocial factors such as living alone, living in rural area, perceived stigma, medical illness, family history of suicide and depressive symptoms were associated with suicidal behavior.

Policy implications in the community health care may be benefits from launching preventive strategies such as improve standardized screening for depression and perceived stigma among individuals with problematic substance uses are recommended to prevent suicidal behavior in the community.

## Data Availability

The datasets used and/or analyses during the current study are available from the corresponding author on reasonable request.

## Abbreviations

CAGE: Cut down, Annoyed, Guilty, and Eye-opener
CI: Confidence Interval
DALYs: Disability-Adjusted Life Years
PHQ: Patient Health Questionnaire
SBR-Q: Suicidal Behavior Questionnaire -Revised
SPSS: Statistical Package for Social Science
USA: United State of America
WHO: World Health Organization
